# Emerging role of the Hippo pathway in autophagy

**DOI:** 10.1038/s41419-020-03069-6

**Published:** 2020-10-20

**Authors:** Dongying Wang, Jiaxing He, Bingyu Huang, Shanshan Liu, Hongming Zhu, Tianmin Xu

**Affiliations:** Department of Obstetrics and Gynecology, The Second Hospital, Jilin University, 218 Zi Qiang Street, Changchun, Jilin, 130000 China

**Keywords:** Macroautophagy, Cancer

## Abstract

Autophagy is a dynamic circulatory system that occurs in all eukaryotic cells. Cytoplasmic material is transported to lysosomes for degradation and recovery through autophagy. This provides energy and macromolecular precursors for cell renewal and homeostasis. The Hippo-YAP pathway has significant biological properties in controlling organ size, tissue homeostasis, and regeneration. Recently, the Hippo-YAP axis has been extensively referred to as the pathophysiological processes regulating autophagy. Understanding the cellular and molecular basis of these processes is crucial for identifying disease pathogenesis and novel therapeutic targets. Here we review recent findings from *Drosophila* models to organisms. We particularly emphasize the regulation between Hippo core components and autophagy, which is involved in normal cellular regulation and the pathogenesis of human diseases, and its application to disease treatment.

## Facts

- The Hippo pathway and autophagy have complex and reciprocal interactions. These bidirectional links coordinate the autophagic flux to the overall microenvironmental signal and regulate homeostasis and tumorigenesis.
- Autophagy is involved in the biological effects of the Hippo pathway and vice versa. Hippo-mediated modalities profoundly influence autophagic flux and are extensively involved in the intracellular quality control, tissue homeostasis, regeneration, development, and differentiation.
- The association between the Hippo pathway and autophagy is relevant to the pathogenesis of a wide range of human diseases, from metabolic and neurodegenerative diseases, cardiovascular diseases to a variety of human solid tumors.
- The emerging link between the Hippo pathway and autophagy means that targeting the Hippo-YAP-autophagy axis may provide new insights to prevent or promote autophagy in a variety of contexts, influencing metabolic reprogramming, cellular mechanical signals, mitochondrial quality control, and YAP/TAZ transcriptional activity.

## Open questions

- How are the Hippo pathway and autophagy interconnected, and what are the implications of these bidirectional links in homeostasis and tumorigenesis?
- How prevalent is the Hippo-autophagy axis and what is its significance in disease development?
- Will the emerging link between the Hippo pathway and autophagy make the role of targeted autophagy in cancer therapy clearer?

## Introduction

**Fig. 1:**
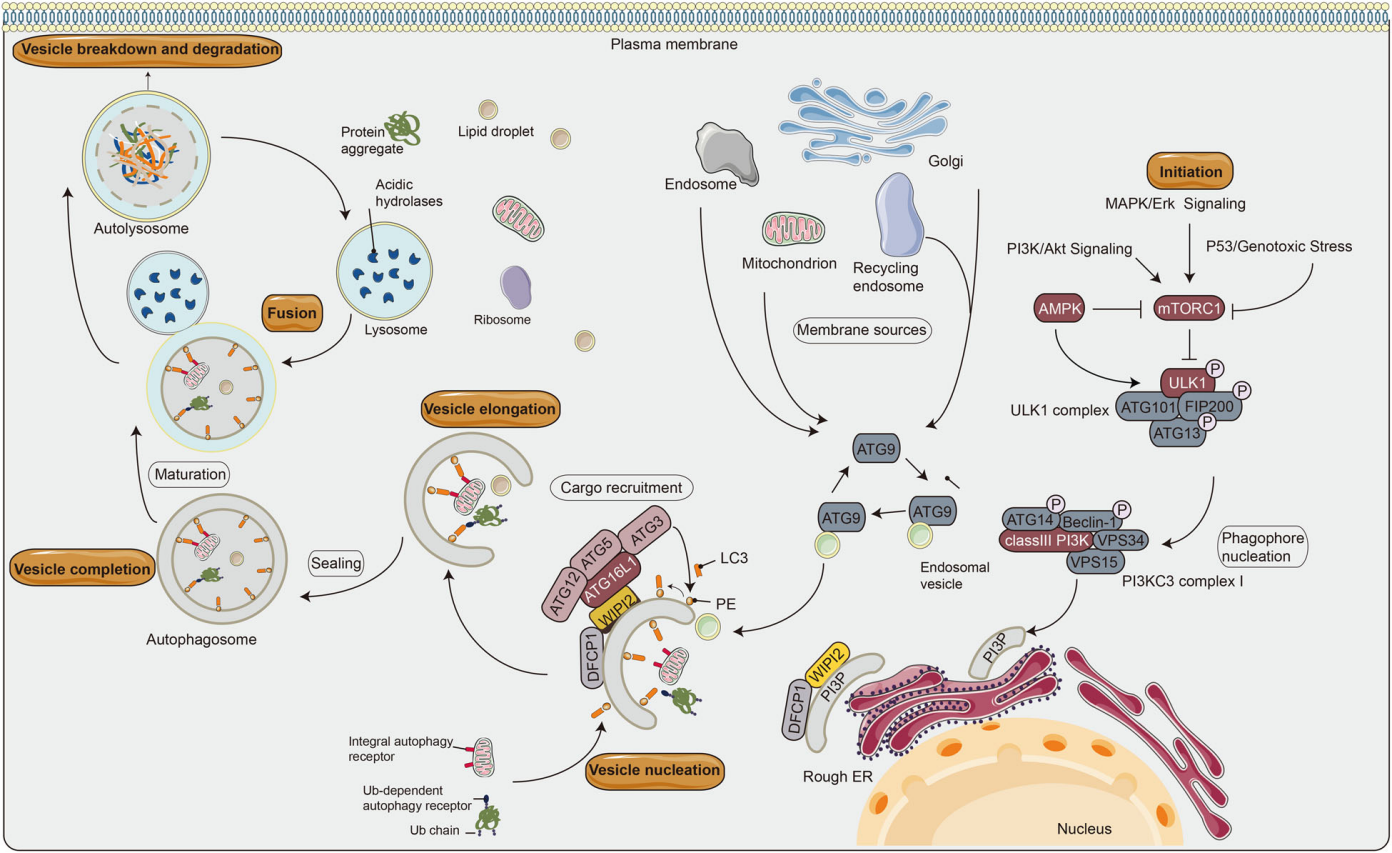
Schematic diagram of the autophagy process in mammalian cells. The mTOR complex 1 (mTORC1) contributes to the initiation of autophagy, integrates upstream signals such as PI3K/Akt pathway, AMPK, P53, and Bcl-2 protein family, which play different regulatory roles in autophagy^135^. The ULK1 complex induces vesicle nucleation and translocates to a characteristic endoplasmic reticulum (ER) structure called omegasome, where it phosphorylates PI3KC3 complex I to produce phosphatidylinositol-3-phosphate (PI3P) in omegasome. Specifically, Beclin1, a Bcl-2-homology (BH)-3 domain-only protein, is phosphorylated by ULK1 and acts as a scaffold for the PI3KC3 complex I, which facilitates localization of autophagy proteins to the phagophore. Atg9 is a transmembrane protein, which participates in the early stage of phagophore formation. PI3P recruits specific autophagy effectors, such as WIPIs (mammalian homolog of yeast Atg18) and zinc finger FYVE-type containing 1 (DFCP1). WIPIs directly binds to ATG16L1 under the regulation of the ubiquitin-like conjugation system to form the ATG12-ATG5-ATG16L1 complex and LC3 (mammalian homolog of yeast Atg8)-phosphatidylethanolamine (PE) binding. Ultimately, the isolation membrane is elongated and closed to form the autophagosome^136^. This binding reaction results in the conversion of LC3-I to LC3-II, a common autophagosome marker. When the autophagosome matures, it sheds the ATG proteins and fuses with the lysosome to produce autophagolysosome. Both the inner membrane of the autophagic vesicle and the luminal contents are degraded by lysosomal hydrolases (cathepsins B, D, and L). The resulting monomer molecules (such as amino acids and lipids) are recycled into the cytoplasm for reuse^137^. The pointed and blunt arrowheads indicate activation and inhibitory interactions, respectively. Ub, ubiquitin.

Autophagy (also known as macroautophagy) is an accommodative process that occurs in different forms of cell stresses, including starvation, hypoxia, infection, high reactive oxygen content, and endoplasmic reticulum stress^1^. During this process, cells capture intracellular proteins and organelles, and transport them to lysosomes for degradation and export the products of autophagic degradation from lysosomes to the cytoplasm for recycling^2,3^. The first genetic screening of autophagy was conducted in Ohsumi’s laboratory, which analyzed this process in yeast and identified 15 autophagy-related proteins (ATGs)^4^. So far, over 30 ATGs have been identified^5^. The autophagy pathway is frequently divided into various individual stages: initiation, vesicle nucleation, elongation of the autophagy membrane, fusion with lysosomes, and degradation of intravesicular products (Fig. 1)^6^. Previously, autophagy has been considered a non-selective process. Recent studies have shown that autophagy can selectively eliminate harmful cytosols such as invading pathogens, dysfunctional organelles, and protein aggregates (called selective autophagy, including lipophagy, mitophagy, xenophagy, and aggrephagy), thereby contributing to the protection of cells in various environmental and metabolic stress^3,7^. Autophagy is strongly associated with neurodegeneration, cancer, metabolic diseases, immune and heart diseases, especially the role of autophagy in cancer^7^. Autophagy plays a dual role in cancer. Under tumorigenesis pressure, autophagy can clear oncogenic protein substrates and toxic unfolded proteins, inhibiting tissue damage and genomic instability^8–10^. Conversely, after tumor formation, increased autophagic flux often allows tumor cells to survive and grow^9,11^. This makes autophagy an interesting target for pharmacologists and clinicians.

**Fig. 2:**
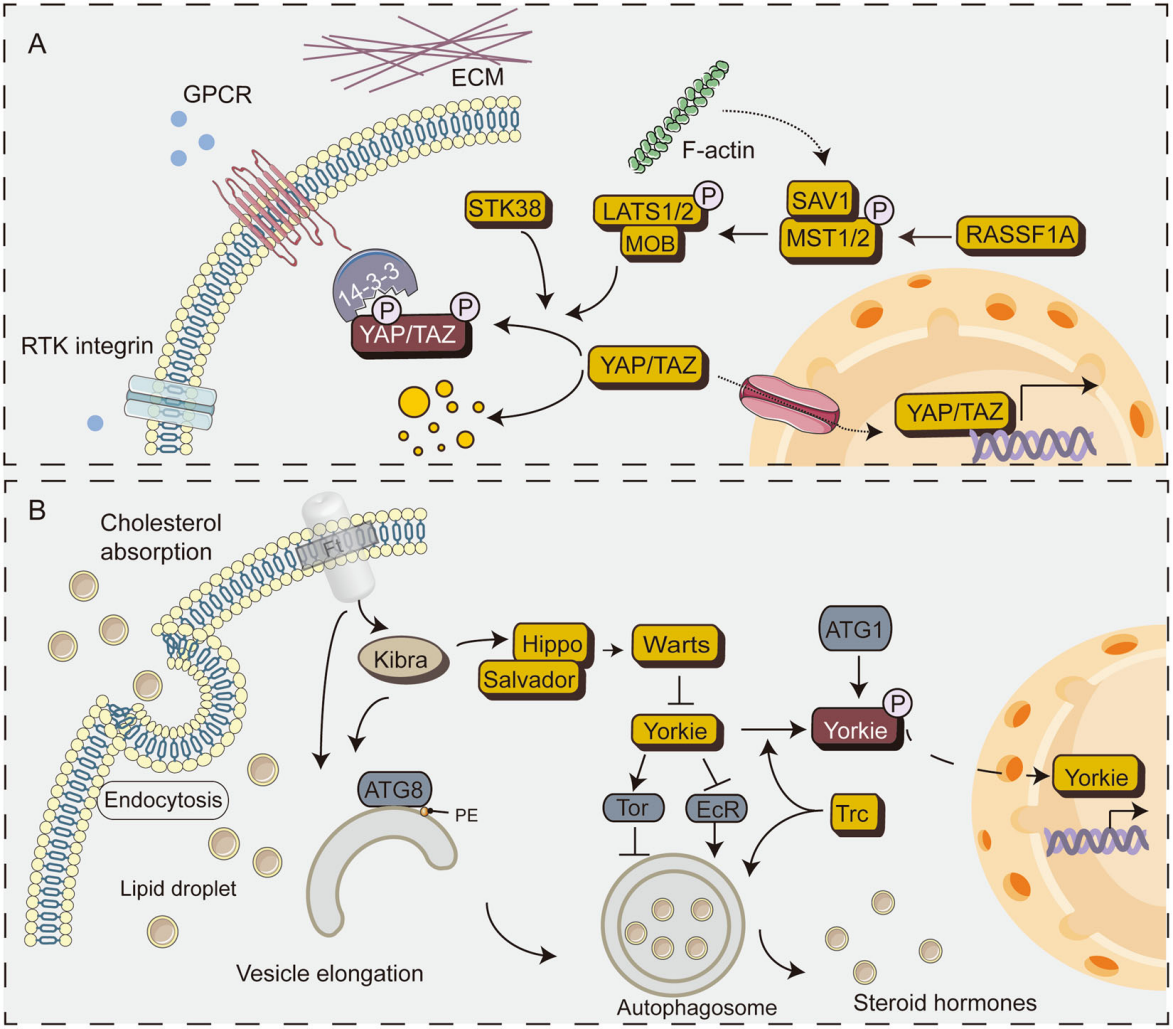
Schematics diagram of the Hipoo pathway in mammals and the crosstalk between the Hippo pathway and autophagy in *Drosophila*. **A** Schematics diagram of the Hippo pathway. In mammalian cells, phosphorylation of MST1/2 activates LATS1/2, which then phosphorylates YAP/TAZ at different Ser residues. Notably, STK38 can directly phosphorylate YAP. Phosphorylated YAP/TAZ is inhibited mainly through two mechanisms: (i) cytoplasmic retention through 14-3-3 binding and (ii) proteasome degradation. Inversely, inhibition of Hippo kinase leads to nuclear accumulation of YAP/TAZ, which bind to TEADs and other transcription factors. **B** Schematic diagram of the crosstalk between the Hippo pathway and autophagy in *Drosophila*. Typically, autophagy inhibits overgrowth of epithelial tissue. When the Hippo function is reduced, this mechanism is restricted. Atg1 phosphorylates Yorkie in a Hippo-Warts-independent manner, blocks the binding of Scalloped (TEADs in mammals) and decreases the activity of Yorkie. In addition, Warts (LATS1/2 in mammals) regulates autophagy via the EcR and Tor pathways. Trc (NDR1 in mammals) promotes the formation of autophagosome. Furthermore, Kibra (WWC1/2 in mammals) and Ft (FAT1-4 in mammals) are novel autophagy-regulated genes that promote ATG8-mediated elongation of the isolated membrane. The Ft mutant increases autophagy flux. The pointed and blunt arrowheads indicate activation and inhibitory interactions, respectively. Abbreviations: EcR, ecdysone receptor; Tor, target of rapamycin kinase; Trc, tricornered.

The Hippo-Yes-associated protein (YAP) pathway is an evolutionarily conserved pathway that controls organ size and tissue homeostasis^12^. The core kinase cassettes of the mammalian Hippo-YAP pathway consist of the mammalian sterile 20-like protein kinase 1 (STK3/MST2 and STK4/MST1) and an adapter protein, salvador family WW domain-containing protein 1 (SAV1)^13,14^, which may phosphorylate and activate the large tumor suppressor kinase 1/2 (LATS1/2)^15^. Adapter protein MOB kinase activator 1A (MOB1A) and MOB1B are also involved in the phosphorylation process^16^. YAP and PDZ-binding motif (TAZ, also known as WW domain-containing transcription regulator 1) are the major downstream transcription coactivators of the Hippo pathway. The phosphorylation of YAP/TAZ by the upstream kinase cascades MST1/2-LATS1/2 promotes the interaction of YAP and TAZ with cytoskeletal proteins, retains YAP and TAZ in the cytoplasm, and prevents their importation into the nucleus for transcriptional activation^15,17^. In contrast, when dephosphorylated, YAP can enter the nucleus and bind to the transcription factor TEA domain family member (TEADs) to control the expression of target genes^15,18^. YAP and TAZ rapidly shuttle between the nucleus and the cytoplasm by complex upstream components. LATS1/2-mediated phosphorylation limits the rate at which YAP and TAZ are imported into the nucleus. In addition, tethering of YAP and TAZ to the cytoskeletal proteins inhibit them as cellular mechanotransduction receptors^19^. The NDR (nuclear Dbf2-related) protein kinase family, including NDR1/STK38 (Serine/Threonine Kinase 38) and NDR2/STK38L (Serine/Threonine Kinase 38 Like), has identified additional kinases of Hippo signaling, similar to the LATS1/2 status in the Hippo signaling pathway (Fig. 2A)^20,21^. It is established that the Hippo-YAP pathway is regulated by cell–cell contact, cell polarity, cellular mechanotransduction, and G protein-coupled receptor ligands. However, recent studies have shown that autophagy has a series of crosstalk with the Hippo-YAP pathway. In physiological settings, the two conserved pathways, autophagy and Hippo-YAP signaling are essential in the protection of homeostasis. It has been shown that the deletion of autophagy-related genes interacting with the Hippo kinase cascades is associated with an accrued propensity of laboratory animals to spontaneously develop various disorders (Table 1). In this review, we summarize the regulation of autophagy by the Hippo-YAP pathway and discuss the multidisciplinary function of Hippo-YAP-autophagy in cells and various disorders.

**Table 1:** The core components of Hippo pathway affect various disorders via autophagy.

Author, year	Disorders	Experimental models	Effector cell	Effects
Zhang et al.^45^ Lin et al.^138^ You et al.^139^	DCM	Streptozotocin induce experimental diabetes in mice	CMEC/cardiomyocyte	MST1 knockdown upregulated autophagy and prevented apoptosis in cardiomyocytes and CMEC.
Shi et al.^140^	DCM	Streptozotocin constructed diabetic model in endothelium-specific MST1 Tg mice	CMEC/cardiomyocyte	The MST1-enriched exosomes released from CMECs inhibit autophagy and glucose metabolism, thereby promote apoptosis in cardiomyocyte.
Yuan et al.^141^	Atherosclerosis	ApoE^−/−^ mice	HUVECs	Laminar flow protects the endothelium, inhibits Hippo-YAP signaling by promoting endothelial autophagy and SIRT1 expression, and blocks the formation of atherosclerotic plaques.
Wang et al.^142^	Atherosclerosis	ApoE^−/−^: Mst1^−/−^ and ApoE^−/−^: Mst1 Tg mice	Murine macrophage	In ApoE (^−/−^) mice, MST1 may stabilize atherosclerotic plaques by inhibiting macrophage autophagy and promoting macrophage apoptosis.
Shang et al.^143^	Septic cardiomyopathy	Lipopolysaccharide (LPS)-induced septic cardiomyopathy MST1^−/−^ mice	Cardiomyocyte	Septic cardiomyopathy is characterized with MST1 upregulation and deletion of MST1-activated mitophagy, thereby attenuated LPS-mediated mitochondrial damage.
Yu et al.^144^	Cardiac I/R injury	Mst1^−/−^ mice	Cardiomyocyte	MST1 deficiency activates protective mitophagy, thereby reducing cardiomyocyte mitochondrial apoptosis and regulating mitochondrial homeostasis.
Yao et al.^145^	Hypertension	Infusion of Ang II induces hypertension in mice	HUVECs	In endothelial cells, mTORC1 regulates autophagy-dependent YAP degradation and controls blood pressure via COX-2/mPGES-1/PGE 2 cascade.
Lee et al.^146^	ALS	ALS mouse model	Mouse motor neuron-like NSC34 cells	The activation of MST1 by SOD1 leads to autophagosome accumulation and blocking autophagy flux, which contribute to the demise of motor neurons both in vitro and in vivo.
Zhang et al.^147^	SCI	MST1^−/−^ and MST1 Tg SCI-induction mice	—	MST1 deficiency promotes posttraumatic spinal motor neuron survival via enhancement of autophagy flux.
Hsu et al.^98^	Barth syndrome	—	MEFs	TAZ deficiency in MEFs caused defective mitophagosome biogenesis (the mitophagy in mitochondria quality control) and leads to impaired oxidative phosphorylation and oxidative stress.
Liang et al.^70^	TSC	TSC mouse model	Mouse embryonic fibroblast	YAP is upregulated by mTOR in mouse and human perivascular epithelioid cell tumors (PEComas), and autophagy impairs YAP degradation in TSC-deficient cells, suggesting that the regulatory effects of YAP by mTOR and autophagy are therapeutic targets.
Xiao et al.^148^	Doxorubicin-induced cardiotoxicity	DOX-induced cardiotoxicity model in mice	Rat cardiomyocytes	YAP/Parkin pathway presented DOX-induced cardiotoxicity in mouse heart by enhancing mitophagy.
Zhou et al.^149^	NAFLD	MST1^−/−^ and MST1 WT NAFLD mouse model	Mouse primary hepatocytes	MST1 deletion reversed Parkin-related mitophagy, suppressed hepatocyte mitochondrial stress, prevented diet-induced NAFLD.
Li et al.^49^	HCC	Induction of HCC by intraperitoneal injection of diethylamine (DEN) in wild-type and RASSF1A-knockout mice.	Mouse primary hepatocytes	RASSF1A inhibits PI3K-AKT-mTOR pathway through MST1 to enhance autophagic flux, further inhibiting HCC and improving survival.
Li et al.^92^	HCC	Induction of HCC by intraperitoneal injection of diethylamine (DEN) in wild-type and liver-specific LRPPRC-knockout mice.	Mouse primary hepatocytes	LRPPRC acts through YAP-P27 to control cell ploidy and P62 hence regulating autophagy maturation.
Lee et al.^88^	HCC	Liver-specific Atg7-knockout mice Atg7/YAP double-knockout mice	The murine and human hepatocyte lines	Atg7 knockdown suppressed autophagy and YAP nuclear localization. YAP acts as an autophagic substrate in liver differentiation and carcinogenesis.
Liu et al.^150^	PTC	Clinical thyroid papillary carcinoma tissue microarray analysis	PTC cell lines	In papillary thyroid cancer, YAP expression correlates with clinicopathological parameters. In vitro, YAP inhibits autophagy but enhances cell proliferation*.*
Li et al.^151^	Breast cancer	Human breast tissue microarray; MCF-7 cells were subcutaneously injected into BALB/c athymic nude mice	Breast cell line and breast cancer cell line	HBXIP inhibits MST1 acetylation, leading to autophagy-dependent degradation of MST1, HBXIP-mediated reduction of tumor suppressor MST1 promotes the growth of breast cancer cells in vitro and in vivo.
Yan et al.^69^	Gastric cancer	—	Normal gastric mucosal cell line and gastric cancer cell line	Knockdown of YAP causes mitochondrial apoptosis and cellular oxidative stress, which subsequently inhibits mitophagy, cancer cell survival, and migration.
Wang et al.^89^	Lung cancer	Lung cancer and adjacent normal tissues	Lung cancer cell line	Aurora A upregulates YAP expression by blocking autophagy and Aurora A kinase expression is positively correlated with YAP.
Zhang et al.^152^	Esophageal cancer	—	Esophageal cancer cell line	MST1 overexpression inhibits mitophagy activity, augments IL-24-induced esophageal cancer death via enhanced mitochondrial stress.
Fan et al.^153^	Multiple myeloma	PINK1-knockout mice and C57BL/6 WT controls Myeloma xenograft mouse model	Multiple myeloma cell line	Activation of PINK1-dependent mitophagy inhibits migration, suppresses myeloma cell homing to calvarium, and decreases osteolytic bone lesions via the MOB1B-mediated Hippo-YAP/TAZ pathway.
Hu et al.^154^	Pancreatic cancer	—	Normal ductal epithelial cell line and pancreatic cancer cell line	MST1 upregulation regulates pancreatic cancer cell apoptosis through mitofusin 2 (Mfn2)‑mediated mitophagy.
Wei et al.^155^	Colorectal cancer	Colorectal cancer xenograft mouse model	Colorectal cancer cell line	FAT4 suppresses colorectal cancer by promoting autophagy and inhibiting the epithelial-to-mesenchymal transition (EMT).

*ALS* amyotrophic lateral sclerosis, *ApoE−/−* apolipoprotein E-deficient, *CMEC* cardiac microvascular endothelial cell, *DCM* diabetic cardiomyopathy, *HBXIP* hepatitis B Virus X interacting protein, *HCC* hepatocellular carcinoma, *HUVECs* human umbilical vein endothelial cells, *I/R* ischemia-reperfusion, *MEFs* primary mouse embryonic fibroblasts, *MST1*^*−/−*^ MST1 knockout, *MST1 Tg* MST1 transgenic, *NAFLD* non-alcoholic fatty liver disease, *PINK1* PTEN-induced putative kinase 1, *PTC* papillary thyroid carcinoma, *SCI* spinal cord injury, *SOD1* superoxide dismutase 1, *TSC* tuberous sclerosis complex, *WT* wild type.

## Mechanism of autophagy

Autophagy initiation begins with the activation of the Unc-51-like autophagy activating kinase 1 (ULK1, also known as ATG1) complex, including ULK1, ULK2, RB1 inducible coiled-coil 1 (FIP200), and ATG13. This leads to the recruitment of ATGs to the specific subcellular location called the phagophore assembly site, which activates class III phosphatidylinositol-3-kinase (PI3KC3) complex I, including VPS34, Beclin1 (mammalian homolog of yeast Atg6), p150 (mammalian homolog of yeast VPS15), and Atg14 or ultraviolet radiation resistance-associated gene protein (also known as P63), and nucleation of an annular structure of the isolation membrane, called phagophore^22,23^. The ATG5–ATG12 complex conjugates with ATG16 to expand the autophagosome membrane, causing the phagosome to expand into a sphere. The enzymolysis of Atg4 to LC3 (Atg8 family protein) produces cytoplasmic LC3-I, which conjugates to lipid phosphatidylethanolamine to form LC3-II and then recruits to the autophagosome membrane. Eventually, the autophagosome fuses with the lysosome and the contents are degraded, thereby enabling cellular metabolic pathways and the renewal of specific organelles (Fig. 1)^1,23–26^. Furthermore, p62 (also known as SQSTM1) is an autophagic modifier of the LC3 family that acts as a bridge between LC3 and ubiquitinated substrates^27,28^. p62-bound polyubiquitinated proteins are integrated into the completed autophagosome and degraded in autolysosomes^29,30^. Whereas increased p62 levels are associated with autophagy inhibition, decreased p62 levels are associated with autophagy activation, revealing that the steady-state levels of this protein could reflect the state of autophagy^29–31^. Thus, p62 combined with LC3 completely monitors the autophagic flux^32^.

## Crosstalk between the Hippo pathway and autophagy in *Drosophila*

Hippo signaling is essential for proper growth control in *Drosophila* and the loss of hippo (MST1/2 in mammals) causes tissue overgrowth^33^. Interestingly, autophagy induction actively suppresses hippo-induced tissue overgrowth. Meanwhile, Atg1 overexpression inhibits Yorkie (YAP in mammals), further suppressing epithelial overgrowth and cell proliferation^34^. Mechanistically, Atg1/ULK1 phosphorylates Yorkie at two serine residues, S74 and S97, thereby blocking transcriptional activation and inhibition of Yorkie activity. Atg1-mediated phosphorylation is an additional inhibitory input independent of the Hippo-Warts pathway^35^.

Steroid hormones are critical signaling molecules for growth regulation^36^. Warts (LATS1/2 in mammals) regulate steroid hormone production through an autophagy-dependent pathway (also called lipophagy). Precisely, Warts control the production of *Drosophila* steroid ecdysone through their effector microRNA bantam, which responds to nutrients, thus mobilizing the transport of the steroid precursor cholesterol^37^. Notably, YAP (mammalian Yki homolog) regulates steroidogenesis in tumor cells^38^, indicating that the regulation of steroidogenesis by the Wts-Yki pathway may be an evolutionarily conserved mechanism.

Trc (NDR1 in mammals) acts as a conserved regulator of autophagy and is required for early autophagosome formation in fly larvae^39^. Kibra, upstream components of the *Drosophila* Hippo pathway, act as autophagy regulatory factors required for proper autophagy function^40^. *Drosophila* protocadherin Fat (Ft) is a cell adhesion molecule in the Hippo pathway that regulates growth and planar cell polarity^41^. Ft mutations cause neurodegenerative changes through autophagy defects; autophagosomes accumulate in the Ft mutant photoreceptors, which are filled with partially degraded material and damaged mitochondria^41^. In conclusion, the core components of the *Drosophila* Hippo pathway are involved in the regulation of autophagy at multiple levels and several crosstalks exist in these two conserved pathways (Fig. 2B).

## Regulation of autophagy by the Hippo pathway core kinase cassettes in mammals

### MST1/2 protein kinases

Posttranslational modifications caused by the Hippo pathway kinases have become a powerful means to regulate autophagy in mammals. STK3/MST2 and STK4/MST1 are critical components of the Hippo pathway, which play a pivotal role in organ size control and tumor suppression. Recent studies have shown that STK3/STK4 can also be involved in the regulation of autophagy, which dynamically interacts with the Atg8 family of autophagy proteins in vitro. Specifically, they both phosphorylate LC3 at threonine 50. STK3/STK4-mediated phosphorylation is critical for the fusion of autophagosomes with lysosomes and the ability of cells to clear intracellular cargo (such as bacteria)^42,43^. STK3/STK4 deletion leads to protein aggregate accumulation of autophagic substrates p62 and LC3-II^42,44,45^. STK4/MST1 phosphorylates Beclin1 in its BH3 domain at Thr108, thereby inhibiting the Beclin1–Vps34 complex, which directly inhibits autophagy. Phosphorylation cascade can enhance Beclin1-Bcl-2 interaction and induce apoptosis^44–46^. RASSF1A, a Hippo pathway scaffold protein, binds to MST1, promotes the activation of MST1 and causes apoptosis (induced by the death receptor signaling pathway)^47,48^. The loss of RASSF1A can also lead to the blockage of the autophagic flux^49^. The regulation of autophagy by MST1/2 is involved in several human diseases (see Table 1).

### NDR protein kinases

**Fig. 3:**
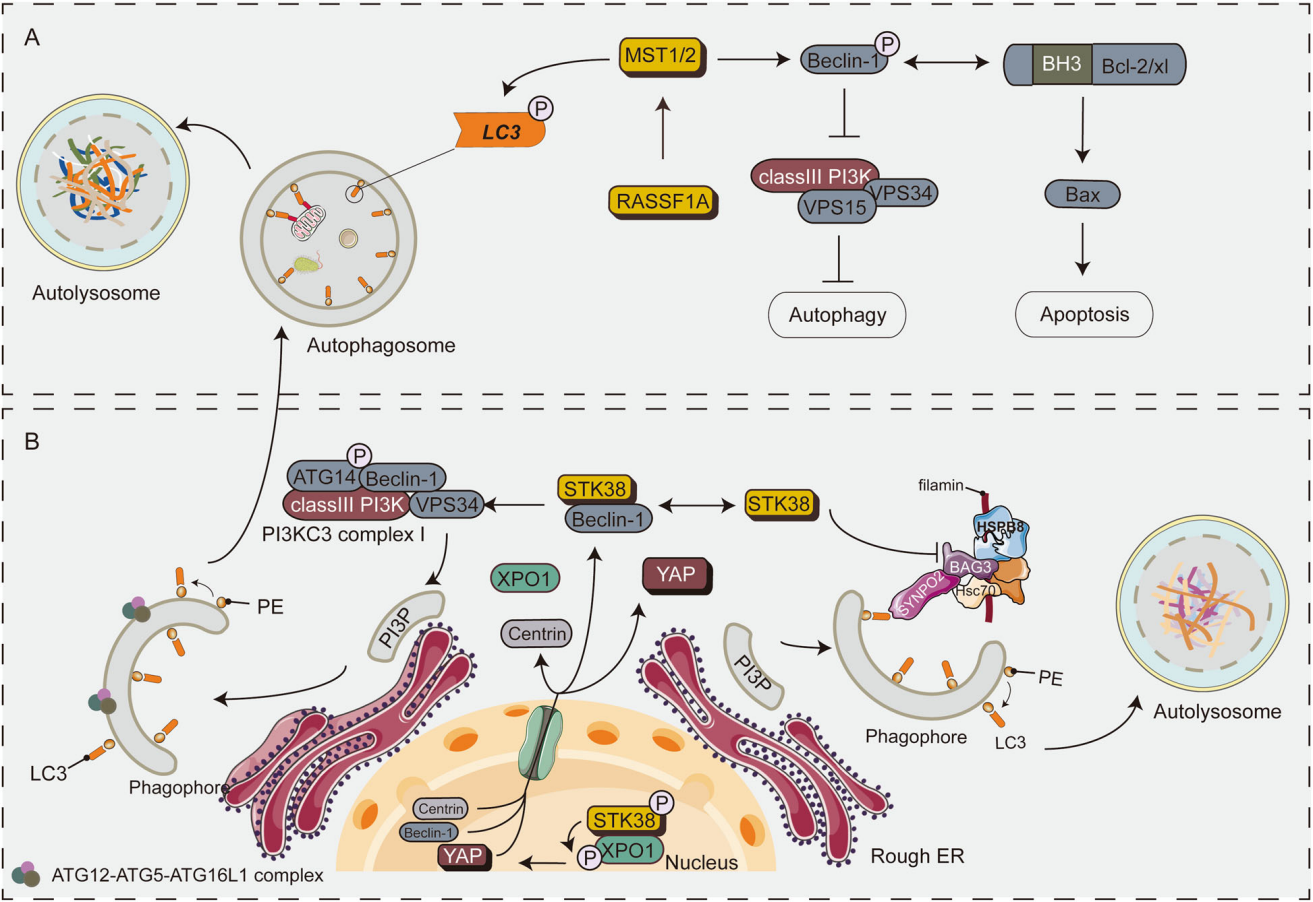
A schematic diagram showing the core components of the Hippo pathway regulating autophagy in mammals. **A** STK3/STK4 kinases are essential for autophagy. Specifically, STK3/STK4 directly phosphorylates LC3 at threonine 50 (Thr50) in mammalian cells, promotes the fusion of autophagosomes with lysosomes and the degradation of cargo in autolysosomes. MST1/STK4 phosphorylates the BH3 domain of Beclin1 at Thr108 and inhibits Vps34 kinase activity, thereby preventing the formation of autophagosome. RASSF1A promotes the initiation and maturation of autophagy by regulating MST1. In addition, MST1 mediates the interaction between Beclin1 and Bcl-2 thereby inducing apoptosis. **B** STK38 phosphorylation of XPO1 on S1055 is vital for the nuclear export of crucial intracellular signal sensors such as Beclin1, YAP1, and Centrin1. Cytoplasmic STK38 interacts with Beclin1 and promotes the formation of the Beclin1-ATG14-Vps34 complex, leading to the formation of PI3P. CASA is activated in mechanically stressed cells and tissues under the regulation ofSTK38. STK38 disrupts the interaction of BAG3 with HSPB8 and SYNPO2. Moreover, CASA activation is independent of the STK38 targets BECN1.

NDR1/2 (STK38/STK38L) is regulated through alterations in the subcellular localization and phosphorylation status, which influence cell cycle, apoptosis, and autophagy in mammalian cells^21^. Furthermore, STK38/STK38L acts as a major stress response and plays an essential role in autophagy. Precisely, STK38 regulates itself and XPO1 (exportin-1) nuclear export by phosphorylating XPO1 on serine 1055, thereby supporting autophagy regulator Beclin1 and Hippo effector YAP shuttle into the cytoplasm^50^. STK38 is also a new binding of Beclin1, which promotes autophagosome formation in mammalian cells. Conversely, STK38-depleted cells reduced PI3KC3 complex I (Beclin1-ATG14-Vps34) and PI3P formation, resulting in reduced autophagosome formation^39^. Moreover, STK38 regulates the chaperone-assisted selective autophagy (CASA), which initiates the CASA complex (including Hsc70, HspB8, synaptopodin-2 (SYNPO2), and the co-chaperone BAG3) and mediates the degradation of misfolded, damaged, and aggregation-prone proteins^51^. STK38 further inhibits BAG3-mediated autophagy in a kinase activity-independent manner, which relies on the remodeling of BAG3 chaperone complexes and disrupts the interaction of HspB8 and SYNPO2^52^. The underlying mechanism by which Hippo pathway core kinase cassettes regulate autophagy is shown in Fig. 3.

## Crosstalk between transcriptional coactivators YAP/TAZ and autophagy

**Fig. 4:**
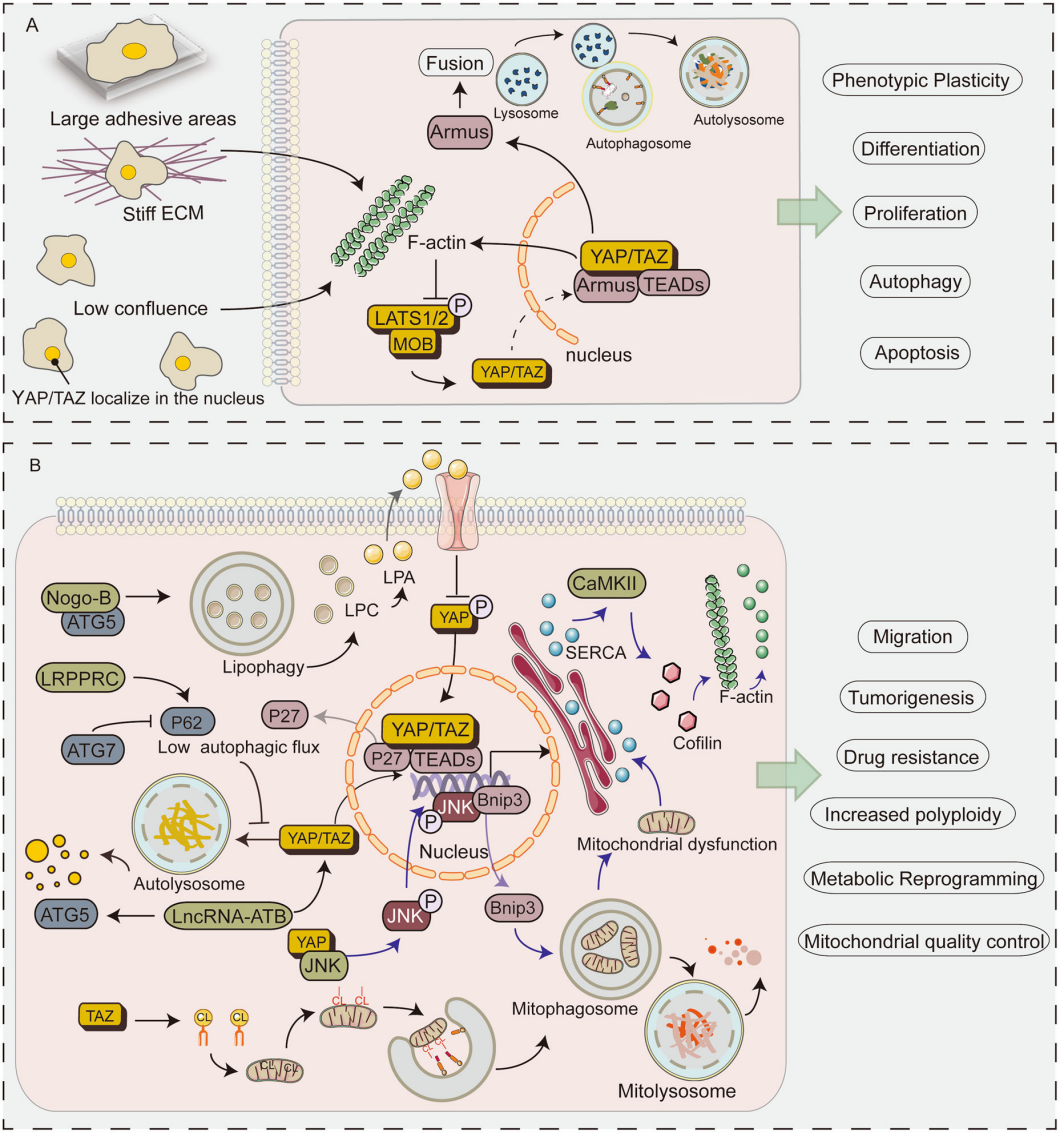
Schematic diagram showing the role of YAP and TAZ in autophagy. **A** When cells are at low density and on a stiff extracellular matrix (ECM), F-actin level is elevated leading to activation and nuclear import of YAP/TAZ, and upregulation of YAP/TAZ targets (such as myosin II and Armus). Activation of YAP/TAZ promotes F-actin accumulation. Cell mechanics control autophagic flux by regulating the transcriptional activity of YAP/TAZ. The YAP/TAZ-autophagy axis regulates a series of biological processes, such as proliferation, apoptosis, differentiation and phenotypic plasticity. **B** Loss of Atg7 or LRPPRC decreases autophagic flux. As an autophagic substrate, YAP cannot be degraded by autophagy, which increases nuclear localization of YAP. Activated YAP triggers accumulation of p27, which in turn leads to cellular polyploidy. lncRNA-ATB influence autophagy by participating in the transcriptional regulation of ATG5. In addition, lncRNA-ATB promotes autophagy by regulating YAP activation. Nogo-B interacts with ATG5 to promote lipophagy leading to LPC-dependent inhibition of YAP phosphorylation and enhances the oncogenic activity of YAP. YAP promotes metastasis via the mitophagy-SERCA-CaMKII pathways and cofilin/F-actin/lamellipodium axis. YAP binds to JNK in the cytoplasm, inducing JNK phosphorylation and nuclear localization, enhancing Bnip3 transcriptional activity. The Bnip3-induced mitophagy leads to mitochondrial dysfunction and ATP deficiency. Insufficient ATP inactivates SERCA and triggers [Ca2+]_*i*_ overload; [Ca2+]_*i*_ which phosphorylates CaMKII and inactivates cofilin, ultimately leading to F-actin degradation and abrogation of lamellipodium-based migration. Cardiolipin (CL) is a phospholipid found in the inner mitochondrial membrane. TAZ is required for catalyzation of CL. When mitochondria are damaged, cardiolipin is externalized and LC3 contains CL-binding sites to initiate mitophagy, thereby maintaining mitochondrial quality control. CaMKII, Ca/calmodulin-dependent protein kinases ΙΙ; CL, cardiolipin; LPA, lysophosphatidic acid; LPC, lysophosphatidylcholine; SERCA, sarco/endoplasmic reticulum Ca2^+^-ATPase.

Autophagy acts as a downstream regulator of YAP/TAZ. Although YAP/TAZ controls autophagic flux by regulating the degradation of autophagosomes, YAP/TAZ is also essential for the maturation of autophagosomes into autolysosomes^53,54^. The use of autophagy inhibitors or endogenous knockdown of autophagy-related genes (e.g., *ATG7/10* or *ATG16L1*) can inhibit YAP-mediated cell proliferation. Similarly, double YAP/TAZ knockdown and verteporfin (the inhibitors of YAP/TAZ^55^) treatment significantly impaired autophagy^53,54^. These demonstrated that the Hippo pathway maintains autophagy. In YAP/TAZ-activated cells, especially the aggressive solid tumor cells, the autophagic flux may be increased, thereby enhancing proliferation, invasion and metastasis of these cells. The underlying mechanism by which YAP/TAZ regulates autophagy is shown in Fig. 4.

### Contact inhibition

Contact inhibition is a fundamental characteristic of normal cells. However, the loss of contact inhibition is an important feature of cancer cells^56^. The mechanical signals exerted by the physical state of cells in tissues and contact inhibition have recently been linked to the Hippo-YAP axis^18,57^. The decreased proliferation and cell survival resulting from contact inhibition is partly autophagy-dependent^54^. The Hippo pathway serves as the functionality of YAP and TAZ by regulating their subcellular localization and protein levels^58^. At high cell density, YAP/TAZ is redistributed into the cytoplasm and becomes inactive^18,59^, failing to regulate the expression of *myosin II* gene. This results in a drastic reduction in the formation of the F-actin stress fibers, ultimately impairing autophagosome formation^54^. Conversely, at low cell density, YAP/TAZ localizes in the nucleus and becomes active, resulting in increased F-actin formation, thus promoting autophagosome formation. In conclusion, YAP/TAZ regulates autophagy. The inhibition of YAP/TAZ function, caused by high cell density, reduces basal autophagy^54^. These signal crosstalks regulate the autophagy-dependent clearance of aggregation-prone proteins, survival under metabolic stress, as well as cell proliferation and differentiation^54,60^.

### Mechanotransduction

Cells sense microenvironmental factors through a mechanotransduction system. They translate these stimuli into biochemical signals that control cell growth, differentiation, and cancer progression. Notably, YAP/TAZ is the medium for the mechanical cues indicated by the microenvironment^18^. YAP/TAZ is essential in the degradation of autophagosomes in both the steady-state and induced autophagy contexts. Autophagy is a downstream event of the YAP/TAZ mechanotransduction: mechanical signals act as the upstream inputs to control YAP/TAZ-mediated transcriptional activation of the TBC1D family members (such as Armus). This promotes the fusion of autophagosomal vesicles with lysosomes and regulates autophagy efficiency. Similar to the YAP/TAZ knockdown effect, low mechanical signal input slows down the autophagic flux^53^. Overall, mechanical signals can control autophagic flux through the regulation of YAP/TAZ transcriptional activity.

### Cancer stem cell

Autophagy is critical in quality control, remodeling, and metabolic functions of adult and cancer stem cells (CSCs)^61^. Similarly, YAP/TAZ regulates the biological properties of the stem cells during normal organ development and tumorigenesis^62^. The YAP/TAZ-autophagy connection maintains the transformed properties of the tumor cells. It also influences the acquisition of CSC status in benign cells^53^. Increased levels of YAP/TAZ result in the transformation of terminally differentiated epithelial cells (such as primary mammary gland cells) into stem/progenitor cells^63^. These reprogramming events require YAP/TAZ-dependent regulation of autophagy. YAP expression greatly increases autophagosome clearance in induced differentiated cells, whereas inactivated autophagy-related genes impair the YAP-mediated reprogramming steps^53^. These data suggest that YAP/TAZ requires an effective autophagic flux to maintain CSC-inducing phenotypic plasticity.

## Apoptosis

YAP overexpressed in multiple human solid tumors and inhibited apoptosis^64,65^. YAP located in the nucleus interacts with p73 and promotes apoptosis in response to DNA damage, suggesting a dual role of YAP on apoptosis^66^. The role of YAP in inhibiting cell apoptosis is at least partially autophagy-dependent. In ovarian and breast cancer cells, YAP knockdown increased cisplatin-induced apoptosis by decreasing autophagy^67,68^. Besides, YAP maintains mitophagy, the selective degradation of mitochondria by autophagy, which can block the caspase-9 apoptotic pathway, contributing to the gastric cancer cell survival and migration^69^. In tuberous sclerosis complex (TSC) 1-TSC2-deficient cells, the autophagic system impairs the degradation of YAP, leading to YAP accumulation. This subsequently causes abnormal proliferation, inducing apoptosis^70^. The interaction between autophagy and YAP is important in the control and modulation of apoptosis and apoptotic thresholds.

### Metastasis

The migration of cancer cells into the circulatory or lymphatic system to form metastases is an extremely complex process in which the Hippo-YAP-autophagy axis is extensively involved^71–73^. Recent functional studies suggest that YAP mediates cancer metastasis via the modulation of actin dynamics^74,75^ and the control of transcriptional activity^76,77^, and along with the long non-coding RNA (lncRNA)-dependent manner^78,79^. Once the cancer cells spread to the systemic circulation and colonize distant organs, autophagic flux is induced to respond to the stressful microenvironments, including hypoxia, nutritional deficiencies and the extracellular matrix detachment^80,81^. F-actin polymerization drives the cellular membrane extension in lamellipodia, leading to cytoskeletal rearrangement, thus promoting migration^74,82^. YAP deficiency promoted the phosphorylation of JNK (c-Jun N-terminal kinases), which activated Bnip3 transcriptional activity and contributed to the Bnip3-required mitophagy. Higher Bnip3 caused mitochondrial dysfunction and ATP shortage, degraded F-actin via SERCA/[Ca2+] i/CaMKII/cofilin axis, and attenuated lamellipodium-based migration^75^. In the triple-negative breast cancer (TNBC) cells, autophagy promoted YAP nuclear localization, promoting TNBC cell migration and invasion^83^.

### Hepatocarcinogenesis

YAP and TAZ are widely activated in human malignancies, which plays a vital role in tumorigenesis and the growth of most solid tumors^84^. For example, overexpression of YAP causes prominent hepatomegaly and induces tumor stem cell attributes, and hepatocarcinogenesis^85,86^. Autophagy maintains hepatic organ size and differentiation and, when autophagy is impaired, YAP is a driver of tissue remodeling and tumorigenesis^87^. In vivo, the liver-specific Atg7-deletion (Atg7 knockout (KO)) mice showed an 8.5-fold increase in the relative liver weight compared to the control mice at three months. Dysplastic nodules appeared at 8 months, whereas hepatocellular carcinoma (HCC) developed at 12 months. Meanwhile, the Atg7/YAP double KO mice attenuated hepatomegaly and hepatocarcinogenesis with significantly lower tumor size and number than the Atg7-KO mice^88^.

As expected, knockdown of Atg7 or Atg5 reduced autophagic flux. Interestingly, shAtg7 or shAtg5 induced nuclear translocation of YAP leading to the activation of TEAD4. Furthermore, YAP colocalized with autophagosomes, so that the cytoplasmic degradation of YAP was at least partially autophagy-dependent^88,89^. As YAP is an essential downstream mediator of tissue remodeling, progenitor cell activation, tumorigenesis, and drug resistance in the autophagy-deficient liver, the concomitant loss of YAP attenuates these abnormalities^88,90^. LRPPRC is a mitochondrion-associated protein^91^. The loss of LRPPRC expression promotes hepatocarcinogenesis^92^. Specifically, the deletion of LRPPRC leads to liver-specific YAP nuclear accumulation and induces accumulation of the cyclin-dependent kinase inhibitor p27, which in turn leads to cell polyploidy^92,93^. Concurrently, the deletion of mitochondrion-associated protein LRPPRC reduces p62 protein levels and impairs autophagic maturation. In vitro, LRPPRC knockdown synergistically enhances the diethylamine-induced genomic instability and hepatocarcinogenesis^92^. The lncRNAs are associated with clinicopathological parameters of HCC and can be used as biomarkers for HCC diagnosis^94^. Precisely, lncRNA-ATB activates the YAP-dependent autophagy and increases the expression of ATG5. The high expression of lncRNA-ATB is associated with poor prognosis and pathological characteristics of HCC^95^. Nogo-B, an endoplasmic reticulum residential protein, is highly expressed and promotes tumorigenesis in HCC. Mechanistically, Nogo-B interacts with ATG5 to encourage droplet lipid degradation and induces lipophagy-mediated oxidized low-density lipoprotein metabolism and subsequent lysophosphatidic acid-stimulated YAP oncogenic activity^96^.

### Mitochondrial quality control

Mitophagy removes damaged mitochondria through autophagy, which is essential for mitochondrial quality control, metabolic homeostasis, and energy supply^97^. Notably, TAZ is required for mitophagy but not autophagosome biogenesis^98^. TAZ is a phospholipid transacylase that catalyzes the remodeling of cardiolipin, a mitochondrial endosomal phospholipid. The redistribution of cardiolipin controls the initiation of mitophagy^98,99^. Mechanistically, TAZ knockdown and inducible TAZ depletion prevent LC3 vesicles from recognizing mitophagosomes, thereby inhibiting mitophagy initiation. This leads to impaired oxidative phosphorylation and oxidative stress. Thus, TAZ is required for the initiation of mitophagy. It is involved in mitochondrial quality control. Mutations of the *TAZ* gene can cause Barth syndrome^98,100^.

## Hippo-YAP-autophagy axis in clinical applications

**Fig. 5:**
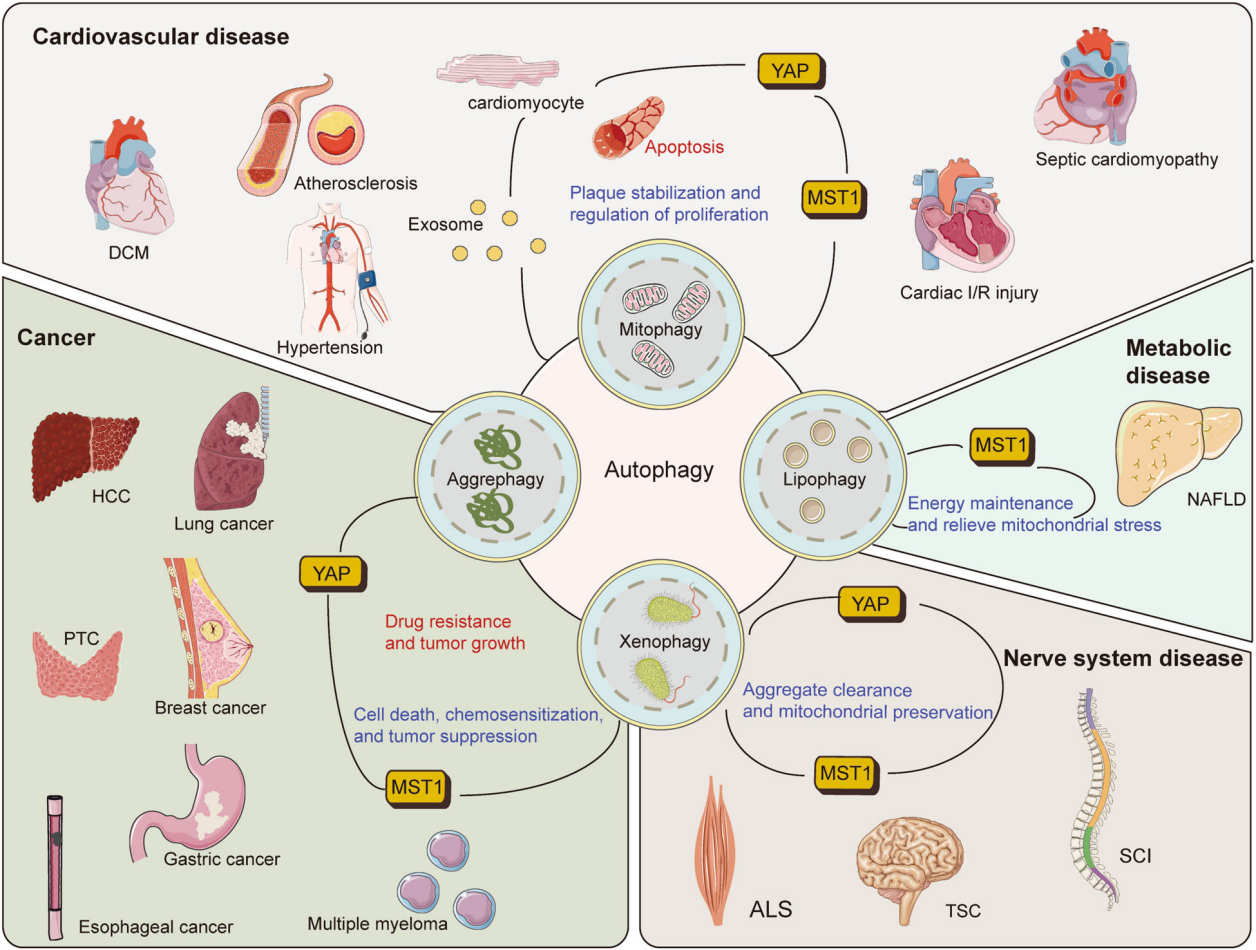
The core components of hippo pathway affect various disorders via autophagy. Hippo-YAP axis regulates autophagy and affects the development of disease progression. Inhibition of disease progression (beneficial process) is shown in blue, whereas promotion of disease progression (harmful process) is shown in red. In many diseases, autophagy clears dysfunctional mitochondria and protein aggregates. As two conserved signaling pathways, the Hippo pathway and autophagy intersect in the regulation of cell death and proliferation, tumorigenesis, and survival and growth of tumor cells. ALS, amyotrophic lateral sclerosis; DCM, diabetic cardiomyopathy; HCC, hepatocellular carcinoma; I/R, ischemia-reperfusion; NAFLD, non-alcoholic fatty liver disease; PTC, papillary thyroid carcinoma; SCI, spinal cord injury; TSC, tuberous sclerosis complex.

Autophagy is an attractive therapeutic target in numerous diseases. As autophagy has a wide correlation with normal homeostasis, targeting it is particularly challenging. In contrast, the role of the Hippo pathway in cancer is widely described. It plays a vital role in tissue renewal and repair. Therefore, targeting the Hippo-YAP-autophagy axis might provide several promising targets. The emerging link between the Hippo pathway and autophagy is now largely implicated in pathophysiological processes, such as cancer, metabolic and neurodegenerative diseases, and cardiovascular diseases (Fig. 5)^101–103^. Here we introduce some small molecules or drugs that target the Hippo core components autophagy regulatory network (Table 2).

**Table 2:** Small molecules or drugs that target the Hippo core components autophagy regulatory network.

Organ		Diseases	Small molecules or drugs	Effects	Reference
	Brain	SAH	Melatonin	Melatonin play a neuroprotective role by regulating the homeostasis between apoptosis and autophagy through the oxygen species (ROS)-MST1 pathway	Shi et al.^134^
		Glioblastoma	Silibinin	Silibinin induced glioblastoma cell apoptosis and autophagy via inhibition of mTOR and YAP.	Bai et al.^110^
	Heart	DCM	Melatonin	Melatonin protects against DCM by increasing autophagy and reducing apoptosis through MST1/Sirt3 signaling	Zhang et al.^131^
		Cardiotoxicity	Adriamycin	HMGB1 is functionally related to YAP and participates in adriamycin-induced cardiotoxicity by upregulating autophagy.	Luo et al.^129^
	Liver	HCC	Sorafenib	Sorafenib promotes autophagy and is the standard treatment for advanced HCC, LATS1 restricts lethal autophagy in sorafenib-induced HCC cells.	Tang et al.^119^
		Hepatic fibrosis	Dihydrotanshinone I	Dihydromorphone I exerts anti-fibrotic effects by blocking the YAP-TEAD2 complex and stimulating autophagy	Ge et al.^123^
	Pancreas	Pancreatic cancer	Neratinib	Neratinib degrades MST4 via autophagy and is essential for the inactivation of YAP/TAZ.	Dent et al.^118^
	Colon	Colon cancer	Curcumin	Curcumin induces autophagy via inhibition of YAP.	Zhu et al.^109^
		Colon cancer	Shikonin	Shikonin effectively suppress colon cancer cell viability and migration, and induces autophagy via inhibiting the activity of YAP.	Zhu et al.^112^

*DCM* diabetic cardiomyopathy, *HCC* hepatocellular carcinoma, *SAH* subarachnoid hemorrhage.

### Cancer

Autophagy is involved in several tumor progression stages, including tumorigenesis, progression, and malignant status maintenance^73^. In the early stages of tumorigenesis, autophagy maintains genome stability by removing oncogenic protein substrates, toxic unfolded proteins, and damaged organelles. This prevents chronic tissue damage, cell damage, and inflammation. Moreover, autophagy inhibits the accumulation of carcinogenic p62 protein aggregates, thereby promoting tumor suppression^104–106^. At an advanced tumor stage, the autophagic flux increases to cope with various environmental pressures, including hypoxia, nutritional deficiencies, DNA damage, metabolic stress, and chemotherapy. This maintains the survival and growth of tumor cells, and promotes tumor invasion and metastasis^9,73,107^. However, when blocking or promoting autophagy at different stages of tumor development, the biological effects on the tumor cell behavior may vary.

As previously mentioned, YAP acts as an autophagic substrate. The expression of YAP protein and YAP target genes is regulated by the autophagic flux^88,89^. Thus, some small molecules that induce autophagy can reduce the oncogenic activity of YAP/TAZ. Curcumin, a natural polyphenolic compound^108^, induces autophagy in colon cancer cells, further inhibiting cell proliferation and YAP expression^109^. Silibinin, a flavonolignan from the seeds of milk thistle, induced glioblastoma cell apoptosis and autophagy via inhibition of mammalian target of rapamycin and YAP^110^. Shikonin is the main bioactive ingredient extracted from the root of *Lithospermum erythrorhizon*^111^, which exerts anti-colon cancer effects similar to silibinin and inhibits YAP activity by inducing autophagy^112^.

As of May 2020, a search for “autophagy and cancer” on ClinicalTrials.gov revealed 72 studies focusing on the inhibition and evaluation of autophagy to improve the clinical prognosis for cancer patients. Targeted drugs either as single agents or in combinations can exert antitumor effects by enhancing both apoptotic and toxic autophagic processes^113^. For instance, neratinib (ERBB1/2/4 inhibitor) enhanced [pazopanib (the kinase inhibitor) + entinostat (histone deacetylase inhibitor)] lethality against sarcoma and other tumor cell types in vitro and in vivo. Specifically, the triplet combination increases the phosphorylation of YAP/TAZ and promotes the conversion of LC3 and expression of Beclin1 and ATG13, which together enhance autophagosome formation^114,115^. The mammalian STK 26/MST4 stimulates ATG4B activity and increases autophagic flux by phosphorylating ATG4B^116^. The MST4–MOB4 complex can disrupt the assembly of the MST1–MOB1 complex by alternative pairing, thereby increasing YAP activity^117^. Neratinib degrades MST4 via autophagy, enhancing LATS1/2 phosphorylation, and is also required for YAP/TAZ inactivation^118^.

Typically, although LATS1 plays a tumor suppressor role in the Hippo pathway, it also exerts a pro-survival function in the HCC cells^119^. Sorafenib (Srf), a multi-kinase inhibitor that promotes autophagy, is the standard treatment for advanced HCC^120,121^. The blockade of LATS1 expression resulted in increased Srf-induced apoptosis and decreased cell viability in vitro, as well as reduced tumor growth in vivo. LATS1 promotes K27-ubiquitination of Beclin1 on lysines K32 and K263, which inhibits autophagy induction and autophagic flux in HCC cells after Srf treatment. In Srf-nonrespondent patient, LATS1 expression is significantly increased, suggesting that LATS1 is a clinically relevant biomarker for Srf sensitivity^119^. The revelation of LATS1 functionally independent of the kinase activity in autophagy regulation requires consideration for targeted LATS1 kinases therapy.

### Non-cancerous diseases

Autophagy plays a pivotal role in protein quality control, especially in maintaining metabolic homeostasis^122^. Dihydrotanshinone I, a natural monomeric compound isolated from *Salvia miltiorrhiza* Bunge, can improve liver function and reduce liver fibrosis. The underlying mechanism is associated with the cytoplasmic retention of YAP, thereby causing downregulation of fibrogenic gene expression, which stimulates autophagic flux and accelerates the degradation of the liver collagen^123^.

Autophagy offers promising targets for the prevention and treatment of cardiovascular diseases^122^. It has been reported that HMGB1, a chromosomal protein, acts as an autophagy sensor and induces autophagy after prolonged cellular stress^124,125^. The expression of HMGB1 is highly correlated with YAP activity, which is involved in tumorigenesis and acquisition of the tumor stem cell characteristics^126,127^. Adriamycin, an anthracycline chemotherapy drug, can also cause cardiotoxicity^128^. Specifically, adriamycin upregulates HMGB1 expression and induces cardiomyocyte autophagy followed by cardiac damage, whereas YAP reverses adriamycin-induced cardiac damage by downregulating HMGB1^129^. Melatonin regulates autophagy and has both chronobiotic and cytoprotective properties^130^. Melatonin significantly alleviates left ventricle remodeling and cardiac dysfunction in dilated cardiomyopathy by inducing autophagy and alleviating mitochondrial dysfunction, which is partially dependent on MST1/Sirt3 signaling^131,132^.

Autophagy is essential for maintaining proteostasis and a healthy mitochondrial pool, especially in maintaining the homeostasis of non-dividing nerve cells^133^. Melatonin plays a cytoprotective role in a variety of neurodegenerative diseases^130^. In subarachnoid hemorrhage-induced rats, melatonin can regulate the homeostasis between apoptosis and autophagy by inhibiting the ROS-MST1 pathway^134^.

## Conclusions and perspectives

The understanding of the regulatory network between the Hippo-YAP pathway and autophagy has gradually been enriched in recent years. In this review, we show that these two highly conserved signaling pathways are widely involved in pathophysiological processes such as apoptosis, cell proliferation, cell differentiation, and metabolism, and can influence the pathogenesis of human diseases. This multidisciplinary view improves our understanding of why these two signaling pathways have been preserved throughout evolution. However, variations in the activity between autophagy and the Hippo-YAP pathway in different tissue types, tumor microenvironments, and disease states are some of the fundamental puzzles yet to be resolved. In addition, the paradoxical effect of autophagy in cancer makes autophagy-targeted therapy in cancer controversial. However, the Hippo pathway dysregulation occurs in a wide range of human cancers. This is essential in the development of novel and more specific drugs. For example, a combination of Hippo pathway-targeted drugs with autophagy inhibitors and inducers may be potential therapies for various human diseases. A better understanding and targeting of the Hippo-YAP-autophagy axis is an auspicious direction.
